# Enteric methane emissions, energy partitioning, and energetic efficiency of zebu beef cattle fed total mixed ration silage

**DOI:** 10.5713/ajas.18.0433

**Published:** 2018-09-13

**Authors:** Sayan Subepang, Tomoyuki Suzuki, Thamrongsak Phonbumrung, Kritapon Sommart

**Affiliations:** 1 Department of Animal Science, Faculty of Agriculture, Khon Kaen University, Khon Kaen 40002, Thailand; 2 Japan International Research Center for Agricultural Science, Tsukuba, Ibaraki 305-8586, Japan; 3 Bureau of Animal Nutrition Development, Department of Livestock Development, Ratchathewi, Bangkok 10400, Thailand

**Keywords:** Digestibility, Energy Balance, Greenhouse Gas, Zebu, Ruminants

## Abstract

**Objective:**

The main objective of this study was to evaluate the effect of different feeding levels of a total mixed ration silage-based diet on feed intake, total tract digestion, enteric methane emissions, and energy partitioning in two beef cattle genotypes.

**Methods:**

Six mature bulls (three Thai natives, and three Thai natives - Charolais crossbreeds) were assigned in a replicated 3×3 Latin square design, with cattle breed genotype in separate squares, three periods of 21 days, and three energy feeding above maintenance levels (1.1, 1.5, and 2.0 MEm, where MEm is metabolizable energy requirement for maintenance). Bulls were placed in a metabolic cage equipped with a ventilated head box respiration system to evaluate digestibility, record respiration gases, and determine energy balance.

**Results:**

Increasing the feeding level had no significant effect on digestibility but drastically reduced the enteric methane emission rate (p<0.05). Increasing the feeding level also significantly increased the energy retention and utilization efficiency (p<0.01). The Thai native cattle had greater enteric methane emission rate, digestibility, and energy utilization efficiency than the Charolais crossbred cattle (p<0.05). The daily metabolizable energy requirement for maintenance in Thai native cattle (388 kJ/kg BW^0.75^, where BW^0.75^ is metabolic body weight) was 15% less than that in Charolais crossbred cattle (444 kJ/kg BW^0.75^).

**Conclusion:**

Our results suggested that the greater feeding level in zebu beef cattle fed above maintenance levels resulted in improved energy retention and utilization efficiency because of the reduction in enteric methane energy loss. The results also indicated higher efficiency of metabolisable energy utilization for growth and a lower energy requirement for maintenance in *Bos indicus* than in *Bos taurus*.

## INTRODUCTION

Energy loss in ruminant livestock through enteric methane emissions is a problem not only because of the impact on climate change but also owing to the considerable effect on animal productivity. Ruminant animals, particularly cattle (*Bos taurus* [*B. taurus*] and *Bos indicus* [*B. indicus*]), produce significant amounts of methane via anaerobic gut digestion. Compared to other ruminants, beef and dairy cattle contribute the most to methane emissions due to their greater body size, energy intake, and population size; they produce 61% of the emissions attributed to all domestic animals [1] and cause a loss of enteric methane energy accounting for 2% to 12% of gross energy (GE) intake. In addition, methane energy loss reduces the efficiency of feed energy utilization and beef cattle productivity [2]. Therefore, the use of feeding strategies to reduce enteric methane emissions is a priority in improving animal productivity and environmental sustainability [3].

Zebu cattle ( *B. indicus*; also referred to in this report as Thai native cattle) and their crossbreeds with European cattle (*B. taurus*) provide the main genotypes for beef cattle populations because they are considered to be well adapted to heat stress, disease, and the low quality feed found in conditions of humid tropical environments. Crossbreeding has been widely adopted to improve growth performance and meat quality, given the advantages for commercialization in the beef industry of genetic heterosis and the complementarity between high productivity and hot climates. We hypothesized that *B. indicus*×*B. taurus* crossbred beef cattle have a higher energy requirement than *B. indicus* purebred zebu beef cattle. This is important because the energy requirement is a function of energetic efficiency that determines the energy supply required to meet production targets [4–6]. Our previous studies have reported that a lower energy is required for maintaining zebu than for European beef cattle [2,7]. However, little research has been undertaken on the energetic efficiency and enteric methane emissions in *B. indicus*×*B. taurus* crossbred beef cattle under tropical humid conditions. In particular, there are no data available comparing the energy balance between Thai native cattle (*B. indicus*) and Charolais crossbred cattle (*B. indicus*×*B. taurus*). Therefore, the objective of this research was to determine the effect of feeding levels on feed intake, digestibility, enteric methane emissions, energy partitioning, and the efficiency of metabolizable energy (ME) utilization in zebu beef cattle fed total mixed ration silage.

## MATERIALS AND METHODS

### Experiment location and animal care

The experiment was conducted at Khon Kaen University Farm Research Station, Khon Kaen province, Thailand (16.46°N 102.82°E; altitude 169 m above sea level). The management of cattle used in the study and all related procedures were performed according to the Guidelines of the Ethics of Animal Experimentation of the National Research Council of Thailand, with permission of the Animal Ethics Committee of Khon Kaen University (Record No. AEKKU23/2557, Reference No. 0514.1.12.2/27).

### Animals, diet, and experimental design

Animals used in the study were three 2.5 year old Thai native bulls and three 2.0 year old Charolais crossbred bulls (50% Charolais×25% Brahman×25% Thai native cattle) with average body weights of 310±12.8 kg (mean±standard deviation) and 369±32.1 kg (mean±standard deviation), respectively. The experiment employed a replicated 3×3 Latin square design, assigning the cattle breed genotype in a separate square with three periods (21 days per period) and three dietary treatment feeding levels (1.1, 1.5, and 2.0 MEm; MEm represents the ME requirement for maintaining beef cattle that is equal to 486 kJ/kg body weight (BW)^0.75^, where BW^0.75^ is metabolic body weight according to The Working Committee of Thai Feeding Standards for Ruminants (WTSR) [6]). The animals were housed individually in pens (2.5×4.5 m), fed at 09:00 and 17:00 each day, and provided with clean drinking water.

The experimental diet was formulated to meet the nutrient requirements for beef cattle [6]: its analyzed chemical composition and feed ingredients are shown in Table 1. The diets were supplied in the form of an ensiled or fermented total mixed ration (FTMR). The FTMR was prepared by mixing a formulated ingredient ratio of 400 kg fresh matter per batch in a horizontal feed mixer (Pak Thong Chai Pasusat, Nakhon Ratchasima province, Thailand), and loading each batch into polyethylene silo bags (1.50×2.20 m, 0.14 mm thick; Sahavanit Industry Co., Ltd., Bangkok, Thailand). The silo bags were tightly packed using a commercial vacuum cleaner/blower (model 1800W VC-910; Imarflex Industrial Co., Ltd., Bangkok, Thailand), and after preparation were stored outdoors at approximately 25°C to 35°C for at least 15 ensiling days.

### Data and sample collection

Cattle were weighed and recorded on the first and last day of each experimental period in the morning (07:30), to determine body weight and metabolic body weight for each feeding level.

Animals were moved to a metabolic cage for measurements of feed intake, digestibility, and respiratory gases. These measurements, which were made according to the method of Schneider and Flatt [8], were completed within six days of each collection period. Samples of both offered and refused feed, feces (1 kg), and urine (500 mL) containing 6 N (Normal) hydrochloric acid solution (to maintain a urine pH of <3) were sampled and weighed each morning for six days, and stored at −18°C until analysis.

Respiratory gas exchange measurements were conducted during the last three days of the metabolic collection. Oxygen consumption and carbon dioxide and methane emissions for each animal were determined according to the method of Suzuki et al [9]. An indirect respiration calorimetry system, consisting of a ventilated head box (width 105 cm×depth 80 cm×height 173 cm) and flow meter with a thermal flow cell (NFHY-R-O-U, Nippon Flow Cell Co., Ltd., Tokyo, Japan), was used to measure and record the flow rate and total air volume. A dual-chamber paramagnetic oxygen analyzer (Servopro 4100 Gas Purity Analyzer, Servomex Group, East Sussex, UK) was used to determine oxygen concentrations in the in- and outflow lines. An infrared gas analyzer (IR200 Infrared Gas Analyzer, Yokogawa Electric Corporation, Tokyo, Japan) was used to measure carbon dioxide and methane concentrations. The gas analyzers were calibrated daily with standard gases (Takachiho Chemical Industrial Co., Ltd., Tokyo, Japan). Calorimetric system recovery tests were conducted using the carbon dioxide injection method, by which a weighed amount of carbon dioxide gas was released into the system.

Energy partitioning, based on energy intake and energy loss through feces, urine, enteric methane, and heat production (HP) was determined according to the protocol of the Agricultural Research Council (ARC) [4]. The average of the antilog of the intercept of the linear regression between the log of HP and ME intake was used to estimate the efficiency of ME utilization for maintenance (k_m_). Energy retained (ER) was calculated by subtracting the HP from ME intake, and the linear regression of ER on ME intake produced the slope assumed to be the efficiency of energy utilization for growth (k_g_) and estimate MEm using ARC [10].

The fermentation profile and pH of the diet was determined using the technique described by Cao et al [11]. Ammonia nitrogen was measured [12] using a spectrometer (T80+ UV/VIS Spectrometer, PG Instruments, London, UK), and volatile fatty acid and lactic acid content in the FTMR samples were determined using gas chromatography (GC-2014, Shimadzu, Kyoto, Japan), according to the method of Porter and Murray [13]. Samples of offered feed, refused feed, and feces were oven-dried at 65°C for 72 h and then ground in order to be passed through a 1 mm screen. AOAC procedures [14] were used to analyze dry feed and feces samples for dry matter (DM), ash, ether extracts, and crude protein (CP), (methods 967.03, 942.05, 920.39, and 984.13, respectively). Neutral detergent fiber (NDF), assayed with heat-stable amylase and expressed inclusively of residual ash and acid detergent fiber (ADF), was analyzed using the method of Van Soest et al [15]. Urine compounds were sampled to determine N content using the Kjeldahl procedure [14]. The GE of the feed, feces, and urine was determined using a bomb calorimeter (IKA Calorimeter System, C 2000 basic, IKA-Werke, Staufen, Germany).

### Statistical analysis

All data were analyzed using the general linear model procedure of SAS [16] according to a replicated 3×3 Latin square design as follows: Y_ijkl_ = μ+S_l_+A_i(l)_+ρ_j_+τ_k_+ɛ_ijkl_, where Y_ijkl_ is the mean response of cattle breed genotype l , cattle i, period j, treatment k; S_l_, the effect of cattle breed genotype (l = 1 to 2); A_i(l)_, the effect of cattle within cattle breed genotype (i = 1 to 6); ρ_j_, the effect of the period (j = 1 to 3); τ_k_, the effect of treatment (k = 1 to 3); and ɛ_ijkl_ is the random residual error. Because the interaction was not statistically significant (p>0.05), it was removed from the model. Linear and quadratic of contrast of the treatment means were estimated [17].

## RESULTS

### Feed intake and digestibility

Feed intake, digestibility, and growth performance are shown in Table 2. Charolais crossbred cattle had a higher daily intake of feed and nutrients (kg DM and g/kg BW^0.75^) than did Thai native cattle (p<0.01). Increasing the feeding level resulted in an increased daily intake of feed and nutrients (p<0.01). Thai native cattle showed higher values for DM and nutrient digestibility than Charolais crossbred cattle (p<0.05), and increasing the feeding level had no significant effect on digestibility of nutrients. Additionally, increasing feeding levels resulted in increases in body weight gain and average daily gain (ADG) (p<0.01), whereas Charolais crossbreeds had a higher growth performance than Thai native cattle (p<0.05).

### Enteric methane emissions

Methane emission data are presented in Table 3. Charolais crossbred cattle emitted greater total enteric methane (L/d and MJ/d) than Thai native cattle but, after correcting for metabolic body weight, no difference was observed (p<0.05; Table 4). The enteric methane emission rate (L/kg DM intake, L/kg organic matter [OM] intake, and L/kg NDF intake; MJ/100 MJ GE intake) in Charolais crossbreeds was significantly less than that in Thai native cattle. Moreover, increasing the feeding level resulted in a significant linear reduction in the rate of enteric methane emissions, from 6.4% to 5.6% (MJ/100 MJ GE intake).

### Energy partitioning

The energy partitioning results, expressed on the basis of metabolic body size and energy utilization of the cattle, are shown in Table 4. Compared to Thai native cattle, Charolais crossbreeds had a higher GE intake and energy loss in feces, urine, and HP. When corrected for metabolic body weight, digestible energy (DE) intake, ME intake, enteric methane production, and energy retention did not differ significantly between Thai native and Charolais crossbred cattle (p>0.05).

Energy (GE, DE, and ME) intake and energy retention increased linearly (p<0.01) as feeding level increased. The proportions of ME to GE and ME to DE in Thai native cattle were higher than in Charolais crossbred cattle. Results for energy utilization (MJ/MJ) showed a linear increase (p<0.01) with increasing ME intake levels.

### Efficiency of metabolizable energy utilization

**Figure 1:**
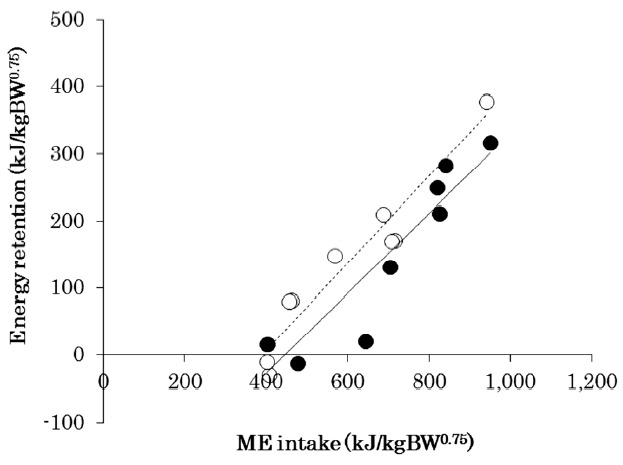
Regression of metabolizable energy (ME) intake on energy retention, scaled for metabolic body weight (kJ/kg BW^0.75^, where BW^0.75^ is metabolic body weight). Thai native cattle (○, n = 9, dashed line): energy retention = 0.65×ME intake–252.0 (R^2^ = 0.92, p<0.001, residual standard deviation = 36.169). Charolais crossbred cattle (●, n = 9, solid line): energy retention = 0.60×ME intake–264.4 (R^2^ = 0.82, p<0.001, residual standard deviation = 50.681).

The result for the regression of ME intake on energy retention was highly significant (Figure 1). The efficiency of k_g_ was 0.65 and 0.60 for Thai native and Charolais crossbred cattle, respectively. The estimated daily MEm was 388.35 kJ/kg BW^0.75^ and 443.68 kJ/kg BW^0.75^ for Thai native and Charolais crossbred cattle, respectively.

## DISCUSSION

In this study, 350-kg big-bag silo storage of FTMR was found to produce good-quality silage that effectively maintained both nutritive and economic values and remained well preserved for more than four weeks. The FTMR fermentation qualities were characterized by a fermentation profile as follows: low pH, VFA and NH_3_-N values and high lactic acid content (Table 1).

In this study, digestibility possibly differed between breeds because of differences between *B. indicus* and its crossbreeds in terms of anatomy, physiology, and the various microbial populations found in the rumen [5]. This finding was similar to the results of a previous study by Cardenas-Medina et al [18], who demonstrated that there was a higher digestibility of DM in *B. indicus* than in *B. taurus*. This may be a result of the higher rate of rumen fermentation in *B. indicus* cattle, due to the different rate at which microorganisms digest cellulose in the rumen. In addition, Ferrell et al [19] make reference to the *B. indicus* breed of cattle utilizing low quality roughage more efficiently than *B. taurus*; these results indicated that the voluntary DM feed intake of beef cattle provided with a tropical feedstuff-based diet is limited to a maximum of 76.3 g/kg BW^0.75^. Feed intake is an important factor to consider because it controls variations in the daily gain of animals [20]. The daily intake of DM and nutrients increases linearly with increasing ME intake levels, resulting in the opportunity for increased growth [2,7].

The present study indicated that feeding level had no effect on nutrient digestibility in cattle. This finding was in contrast to Chaokaur et al [2], who report that the digestibility of DM in Brahman cattle declines significantly with increased feeding level. The ruminal digesta passage rate increases in response to increased feeding levels, reducing the time available for digestion by the rumen microbes. However, the results of the present study were similar to those of Tangjitwattanachai et al [7], who report that the digestibility of all nutrients, except for NDF, shows no significant difference when feeding levels are increased in Thai native cattle. Moreover, our results were in good agreement with Kaewpila et al [21], who has found that an increased feeding level did not significantly affect the digestibility of DM, OM, CP, NDF, and ADF in Thai native cattle fed an FTMR diet, indicating that increasing the feeding level in this diet does not alter the digestibility of DM and nutrients in cattle.

In the present study, the enteric methane emission rate (MJ/ 100 MJ GE intake), the methane conversion factor (Y_m_) were significantly lower in Charolais crossbreeds than in Thai native cattle. Y_m_ value for the two breeds fed an FTMR diet based on good quality tropical feed was near to recommendation by the Intergovernmental Panel on Climate Change [22] (6.5%± 1.0%). Thai native and Charolais crossbred cattle showed no differences in methane emissions (expressed in kJ/kg BW^0.75^). This result was in good agreement with the results of Chuntrakort et al [23], who have determined that methane emissions (kJ/kg BW^0.75^) from Thai native cattle were similar to those of Brahman crossbreeds.

Our results indicated that increasing the feeding level drastically reduced the value of the methane conversion factor and enteric methane emission rate (L/kg DM intake, L/kg OM intake, L/kg NDF intake, and L/kg ADG); this result was similar to other reports [2]. These data highlighted the importance of increasing the feeding level to implement an increase in beef cattle performance without change in the cattle population as a strategy in reducing the impacts of global warming, and in improving environmental sustainability in the tropics.

This study demonstrated that an increase in feeding level improves ME intake, ER, and energetic efficiency (Table 4), due to a decrease in the proportion of energy intake to energy excretion in feces, urine, enteric methane, and HP [2,7,9,10, 24]. This result indicated a strong relationship in the ME and DE ratio, which ranged from 0.80 to 0.88; the proportion of ME to DE recommended by the ARC [4], National Research Council (NRC) [5], and Commonwealth Scientific and Industrial Research Organisation (CSIRO) [25] is 0.81, 0.80, and 0.82, respectively. However, the higher range (0.88) exhibited in the ME to DE ratio in the present study was within the range of 0.84 to 0.88 suggested by the Chaokaur et al [2], and others [7,23].

The NRC [5] have suggested that energy requirement in *B. indicus* is approximately 10% less than in *B. taurus*, while in crossbred cattle (*B. indicus*×*B. taurus*) it is intermediate between the value for the two purebreds. Our study confirmed that MEm in Thai native cattle was lower 14% less than in Charolais crossbred cattle. Variations in MEm may be affected by physiological conditions of breed, sex, age, physical activity, and the temperature of the environment [5,6]. However, the results for MEm in Charolais crossbred cattle (444 kJ/kg BW^0.75^) in the present study were similar to those of Kaewpila et al [21], who reports that MEm in Charolais and Japanese Black crossbred cattle fed in Thailand was 430 kJ/kg BW^0.75^, and 386 kJ/kg BW^0.75^, respectively. CSIRO [25] have also reported that the energy requirement for maintaining *B. indicus* cattle is 20% lower than for *B. taurus*, because the former have a higher heat tolerance genetic potential for production than the latter. This agrees well with Cardenas-Medina et al [18], who report that, in Mexico, *B. indicus* have a 10% lower maintenance energy requirement than *B. taurus*; this reduction with increasing body size may be explained by a lower proportion in the weight of the organs and body protein composition.

Efficiency of metabolisable energy utilization requirement for maintenance (k_m_), the ratio of net energy requirement for maintenance (NEm)/MEm, was used to determine metabolisable energy requirement in beef cattle feeding system worldwide. The results indicated that k_m_ for Thai native cattle (k_m_ = 0.76) was 4% higher than for Charolais crossbred cattle (k_m_ = 0.73), which were within the range (0.36 to 0.81) for beef cattle recommended by Solis et al [26]. Moreover, WTSR [6] suggested that the k_m_ for *B. indicus* (0.64) was higher than the k_m_ for *B. taurus* (0.58). The value of k_m_ may vary according to variation in NEm which varied and influenced by level of feeding, previous plane of nutrition and breed [5]. Garrett [27] suggested that the variation in k_m_ between *B. indicus* and *B. taurus* is affected by body composition and plane of nutrition. The protein turnover may be responsible in variation of k_m_ and that the difference of k_m_ between *B. indicus* and *B. taurus* may explain in part of protein turnover. *B. indicus* have less protein turnover than *B. taurus*, which could explain that *B. indicus* have more efficiency in using MEm than *B. taurus*.

The efficiency of metabolisable energy utilization for growth (k_g_ = 0.65) in Thai native cattle was 5% higher than in Charolais crossbred cattle (k_g_ = 0.60); this is similar to the results of Tangjitwattanachai et al [28], who have determined k_g_ for *B. indicus* (0.51) to be higher than for *B. taurus* (0.45). The values of k_g_ reported in the present study were very close to the findings of Kaewpila [29] who reports k_g_ values of 0.57 and 0.60 for Charolais and Japanese Black crossbred cattle, respectively. The k_g_ value provides an estimate of the partial use efficiency of the ratio of the ME for growth to net energy for growth. Energy can be retained in the form of differences in the percentage of energy retention between protein and fat, related to differences in the efficiency of energy utilization. Garrett [27] reports that the efficiency of ME utilization for protein and fat synthesis ranges from 10% to 40% and 60% to 80%, respectively. Thus, these data could indicate that Thai native cattle have a higher degree of physiological maturity, and consequently show better fat deposition, than Charolais crossbred cattle.

**Figure 2:**
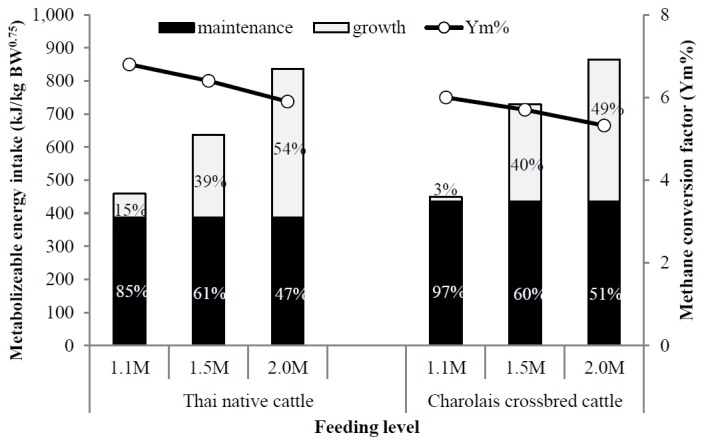
Effect of increasing feeding level (times the metabolizable energy requirement for maintenance [MEm]; M = 388 and 444 kJ/kg BW^0.75^ for Thai native and Charolais crossbred cattle, respectively) on the percentage of metabolisable energy utilization for maintenance and growth to daily metabolizable energy intake (primary horizontal axis, expressed in kJ/kg BW^0.75^) and enteric methane emissions energy to gross energy intake or methane conversion factor (Ym) (secondary horizontal axis).

The effect of increasing the feeding level on ME utilization for maintenance and growth and the enteric methane conversion factor is shown in Figure 2; increasing the feeding level improved the supply of energy available for growth and reduced the methane conversion factor. This result was in good agreement with previous reports [2,7] which have all found that increasing ME intake improves both beef cattle productivity and strategies for enteric methane mitigation [9,20,23,24].

## IMPLICATION

Zebu cattle play an important role in the beef production industry. There is increasing interest in greenhouse gas mitigation strategies to improve the productivity and environmental sustainability of beef production systems. Increasing the feeding level resulted in improved energetic efficiency due to the decreased proportion of energy intake to energy excretion in enteric methane emissions; this therefore improved energy retention in beef cattle fed total mixed ration silage based diet. Methane emissions are not only an important source of greenhouse gases, with major impacts on climate change, but are also a critical factor in the efficiency of feed energy utilization, and are therefore strongly associated with cattle productivity. More feeding trials or on-farm research is needed for the development of a practical and economical zebu crossbred beef farming system.

## Figures and Tables

**Table 1:** Ingredients, analyzed chemical composition, fermentation profile, and energy content of the experimental fermented total mixed ration diet

Items	Diet
Ingredients (% DM)	
Rice straw	20.0
Cassava pulp	30.0
Coconut meal	14.8
Palm kernel meal	25.2
Rice bran	8.5
Urea	0.5
Minerals, mixed1)	0.5
Vitamins, premixed2)	0.5
Total (%)	100.0
Chemical composition (% DM)	
Dry matter	43.1
Organic matter	93.5
Crude protein	11.5
Ether extracts	5.6
Neutral detergent fiber	51.9
Acid detergent fiber	32.5
Fermentation profile	
pH	3.7
Ammonia nitrogen (% of DM)	2.7
Lactic acid (% of DM)	4.1
Acetic acid (% of DM)	1.2
Propionic acid (% of DM)	0.04
Butyric acid (% of DM)	0.02
Energy content (MJ/kg DM)	
Gross energy	17.2
Digestible energy	11.9
Metabolizable energy	10.1

DM, dry matter; MJ, megajoules.

1) Trace minerals premix provided the following per kg concentrate: cobalt, 0.02 g; copper, 1.60 g; iodine, 10.00 g; manganese, 8.00 g; selenium, 0.06 g; zinc, 6.00 g; anti-rancidity, 2.50 g; carrier, 1,000.00 g.

2) Vitamins premix provided the following per kg concentrate: vitamin A, 2,000,000 IU; vitamin D_3_, 4,000,000 IU; vitamin E, 3,000 IU.

**Table 2:** Daily feed intake, digestibility, and body weight in cattle fed a fermented total mixed ration at different feeding levels

Item	Breed		SEM	p-value	Feeding level1)			SEM	p-value2)	
		
	Thai native	Charolais crossbreed			1.1 MEm	1.5 MEm	2.0 MEm		L	Q
Daily feed intake										
Dry matter (kg)	4.2	6.1	0.25	<0.01	4.0	5.5	6.2	0.31	<0.01	0.33
Dry matter (% BW)	1.4	1.6	0.07	0.06	1.2	1.6	1.8	0.83	<0.01	0.41
Dry matter(g/kg BW^0.75^)	58.5	70.6	2.99	0.02	50.0	67.3	76.3	3.66	<0.01	0.39
Nutrient intake (kg/d)										
Organic matter	4.1	5.7	0.24	<0.01	3.7	5.1	5.8	0.29	<0.01	0.33
Crude protein	0.5	0.7	0.03	<0.01	0.5	0.6	0.7	0.04	<0.01	0.34
Neutral detergent fiber	2.5	3.4	0.14	<0.01	2.3	3.1	3.5	0.18	<0.01	0.33
Acid detergent fiber	1.6	2.3	0.09	<0.01	1.5	2.0	2.3	0.12	<0.01	0.34
Digestibility (g/kg)										
Dry matter	709	606	17.4	<0.01	652	681	638	21.3	0.63	0.21
Organic matter	735	641	15.8	<0.01	684	709	671	19.4	0.63	0.21
Crude protein	620	559	16.9	0.03	583	614	571	20.7	0.69	0.18
Neutral detergent fiber	533	426	18.7	<0.01	457	502	479	22.8	0.52	0.36
Acid detergent fiber	395	321	19.1	0.03	357	480	336	23.4	0.54	0.28
Body weight (kg)										
Initial weight	310.1	369.4	3.67	<0.01	344.3	343.5	331.5	4.50	0.08	0.34
Final weight	316.7	385.7	3.12	<0.01	344.3	356.8	352.3	3.82	0.18	0.18
Weight gain	6.6	16.3	3.69	0.03	0.0	13.3	20.8	4.52	0.01	0.61
Average daily gain (kg)	0.3	0.8	0.18	0.03	0.0	0.6	1.0	0.21	0.01	0.61

SEM, standard error of mean; BW, body weight; BW^0.75^; metabolic body weight.

1) MEm, metabolizable energy requirement for maintenance (486 kJ/kg BW^0.75^/d).

2) Polynomial contrast probability of a significant linear (L) and quadratic (Q) effect in the feeding levels.

**Table 3:** Enteric methane emissions from cattle fed a fermented total mixed ration at different feeding levels

Item	Breed		SEM	p-value	Feeding level1)			SEM	p-value2)	
		
	Thai native	Charolais crossbreed			1.1 MEm	1.5 MEm	2.0 MEm		L	Q
Emission rate										
L/d	118.9	149.0	6.03	<0.01	109.7	142.1	150.1	7.38	<0.01	0.22
L/kg DMI	27.6	24.7	0.66	0.02	27.8	26.3	24.4	0.81	0.02	0.85
L/kg OMI	29.5	26.4	0.71	0.02	29.7	28.1	26.1	0.87	0.02	0.85
L/kg NDFI	48.7	43.6	1.12	0.02	49.0	46.4	43.0	1.43	0.02	0.85
L/kg ADG	321.5	263.5	30.59	0.43	638.8	228.1	162.0	17.66	0.02	0.10
MJ/d	4.7	5.9	0.24	<0.01	4.3	5.6	5.9	0.29	<0.01	0.21
MJ/100 MJ GEI	6.3	5.7	0.15	0.02	6.4	6.0	5.6	0.18	0.02	0.85

SEM, standard error of mean; DMI, dry matter intake; OMI, organic matter intake; NDFI, neutral detergent fiber intake; ADG, average daily gain; GEI, gross energy intake.

1) MEm, metabolizable energy requirement for maintenance (486 kJ/kg BW^0.75^/d).

2) Polynomial contrast probability of a significant linear (L) and quadratic (Q) effect in the feeding levels.

**Table 4:** Energy partitioning and utilization in cattle fed a fermented total mixed ration at different feeding levels

Item	Breed		SEM	p-value	Feeding levels1)			SEM	p-value2)	
		
	Thai native	Charolais crossbreed			1.1 MEm	1.5 MEm	2.0 MEm		L	Q
Energy partition (kJ/kgBW^0.75^/d)										
GE intake	1,008.7	1,221.3	49.82	0.02	869.8	1,159.8	1,315.4	61.02	<0.01	0.40
DE intake	725.1	804.7	37.87	0.18	542.6	790.1	962.0	46.38	<0.01	0.52
ME intake	643.7	673.8	36.28	0.57	443.1	683.1	850.1	44.43	<0.01	0.52
Feces excretion	283.6	416.6	27.94	0.01	327.2	369.7	353.4	34.22	0.60	0.50
Urine excretion	18.4	61.9	1.67	<0.01	44.2	37.8	38.4	2.05	0.08	0.20
Methane emission	63.0	69.0	2.68	0.15	55.2	69.2	73.5	3.28	<0.01	0.26
Heat production	459.3	536.7	21.12	0.03	421.5	532.1	540.4	25.87	0.01	0.15
Energy retention	184.4	137.1	36.61	0.39	21.6	151.0	309.7	44.84	<0.01	0.80
Energy utilization										
DE/GE	0.71	0.66	0.019	0.07	0.62	0.68	0.74	0.023	<0.01	0.91
ME/GE	0.63	0.54	0.018	0.01	0.51	0.59	0.65	0.022	<0.01	0.64
ME/DE	0.88	0.83	0.008	<0.01	0.82	0.87	0.88	0.010	<0.01	0.19

SEM, standard error of mean; BW^0.75^, metabolic body weight; GE, gross energy; DE, digestible energy; ME, metabolizable energy.

1) MEm, metabolizable energy requirement for maintenance (486 kJ/kg BW^0.75^/d).

2) Polynomial contrast probability of a significant linear (L) and quadratic (Q) effect in the feeding levels.
